# Editorial of Special Issues “Gut Microbiota–Host Interactions: From Symbiosis to Dysbiosis 2.0”

**DOI:** 10.3390/ijms24108977

**Published:** 2023-05-19

**Authors:** Valentina Zuccaro, Francesca Romana Ponziani, Raffaele Bruno

**Affiliations:** 1 Division of Infectious Diseases I, Fondazione IRCCS Policlinico San Matteo, 27100 Pavia, Italy; raffaele.bruno@unipv.it; 2 Internal Medicine and Gastroenterology, Fondazione Policlinico Universitario Agostino Gemelli IRCCS, 00168 Rome, Italy; francesca.ponziani@gmail.com; 3 Translational Medicine and Surgery Department, Università Cattolica del Sacro Cuore, 00168 Rome, Italy; 4 Department of Medical, Surgical, Diagnostic and Pediatric Science, University of Pavia, 27100 Pavia, Italy

The gastrointestinal (GI) tract is where external agents meet the internal environment. It harbors trillions of bacteria, fungi, viruses, and archaea that constitute the commonly known gut microbiota (GM), which represents a true “superorganism” [1]. In fact, the GM has multiple functions, and is characterized by a dynamic and complex network of interactions with the host. Unsurprisingly, the crosstalk between GM and the host is intense and complex, and depends on host genetics and extrinsic factors such as lifestyle, dietary habits, and medication intake [1]. When dysbiosis occurs, the resulting pro-inflammatory environment can lead to bacterial translocation, systemic immune activation, tissue damage, and cancerogenesis. Additionally, the interplay between the GM and the host must take into account the role of agriculture in preserving biodiversity. In fact, as in humans, the animal GM influences the health of livestock and pets, as well as disease vectors, with consequent implications for human health [2].

Hence, studying GM is a challenge, and an interdisciplinary approach is fundamental to clarifying its role in health and disease, but also to shedding light on unresolved questions in this field. In this new Special Issue, entitled “Gut Microbiota–Host Interactions: From Symbiosis to Dysbiosis 2.0”, of the International Journal of Molecular Sciences, many scientists, with their multidisciplinary effort, have explored the dynamic interplay of the GM with the host and its pathogenetic role in human diseases.

Seven original articles and three reviews have been published, covering multidisciplinary aspects of GM and host interactions.

As already underlined, the study of GM faces several obstacles: first of all is the pathogenic role of some microorganisms in human health. With this in mind, Jirku et al. proposed a study that explored the occurrence of *Dientamoeba fragilis*, a protist neglected because of its apparent minor clinical significance, in the gut of healthy volunteers, but also in the gut of their animals, in order to understand its role in human health and disease. In this study, the key role of lifestyle in GM composition was elucidated, particularly in those living with animals and traveling. However, the prevalence of *Dientamoeba fragilis* observed in healthy volunteers did not allow for the resolution of the scientific debate regarding the commensal or pathogenic role of the protist [3].

Similarly, the scientific community has provided discordant data concerning the beneficial impact as well as the opportunistic and pathogenic role of *Parabacteroides distasonis* on its host. Interestingly, this species has been considered as a new biotherapeutic product. Chamarande et al. aimed to investigate the potential virulence of this species by analyzing strain variability associated with pathogenicity. Several gene clusters encoding different cells surface structures were identified; mutations in these gene sequences might explain the different behavior of different *P. distasonis* [4].

Evaluating the role of the external environment is another important obstacle to studying GM and host interactions. On this basis, Fernandes et al. aimed to analyze the impact of ionizing radiation from radiopharmaceuticals, such as Radium-223 (Ra-223), Iodine-131(I-131), and Technetium-99m (Tc-99m), on the human gut microbiota composition by using fecal samples obtained from healthy volunteers. The experiment demonstrated fold changes in all analyzed taxa with all radiopharmaceuticals, but I-131 was associated with major shifts. Although the experimental design was ex vivo, the work provided a strong basis to guide future studies using more complex models (animal or human ones) [5].

Ecological biodiversity is mainly threatened by global warming, with strong implications for human life; environmental temperature is a recognized critical factor which is able to significantly change the composition of the intestinal microorganisms in insects. Hence, Sun et al. aimed to assess the relationship between these changes and the GM composition of silkworms (*Bombyx mori*) in response to exposure to a high-temperature environment [6].

Moreover, as mentioned above, agriculture plays a crucial role. Livestock farming is a significant source of greenhouse gases such as CO_2_ and CH_4_. Currently, it is well-established that CH_4_ is correlated to the microbial communities of ruminants; despite chickens being the most ubiquitous species of domestic livestock, few studies including data on chickens are available. With this in mind, Cisek et al. aimed to identify the microbial communities involved in the hydrogen sink pathway in chickens in order to evaluate the potential role of dietary manipulation in hydrogen metabolism. This study showed that acetogenesis is the predominant metabolic pathway, and further studies are needed to determine the role of specific gut microbial communities in the hydrogen sink in order to evaluate the impact of dietary manipulation of H_2_ production [7].

Understanding the role and mechanisms of GM in human disease is one of the most appealing issues. Despite the different -omic disciplines that are available nowadays, identifying a link between GM profiles and a particular disease is still challenging. Metagenomics investigations have been extensively applied in the study of GM, and, even if alternative functional omics seem to provide a better interpretation of such a link, they are rarely employed. Type 1 diabetes mellitus (T1D) is an autoimmune disease, the underlying inflammatory mechanisms of which are well-defined. Different factors contribute to T1D pathophysiology, including GM composition: dysbiosis leads to increased intestinal permeability and then to autoimmune attacks on insulin-producing beta-cells, as extensively discussed in the review by Del Chierico et al. [8]. With this in mind, by applying a metaproteomic analysis, Levi Mortera et al. investigated the GM composition of children affected by Type 1 diabetes mellitus (T1D) compared with a group of siblings (SIBL) and a reference control group (CTRL). Authors found a correlation between different GM compositions and the metaproteins implicated in pathways related to inflammation and immune response in the three study groups [9]. Del Chierico et al., in another study, identified GM signatures linked to metabolism and disease markers at the onset of the disease. Specifically, they found that anti-GAD serum levels and acidosis are linked to specific microbial taxa as predictors of progression and severity of T1D. As suggested by the authors, these results could be pivotal in the development of a new treatment approach including probiotics, prebiotics, or fecal microbiota transplantation (FMT) [10].

As mentioned above, dysbiosis seems to have an impact on the process of cancerogenesis. Accumulated knowledge regarding this complex interplay is detailed in the review by Jaye et al. The authors focused on breast cancer and on the potential anticancer activity of some metabolites produced by GM, as well as their potential prognostic role in this setting [11].

Even if the mechanisms underlying cancer development are not fully established, intestinal permeability seems to contribute to this process. Di Tommaso et al. meticulously reviewed the current evidence regarding the “gut-vascular unit”. The collected data demonstrate that gut endothelial cells (ECs) are influenced by GM composition. Consequently, GM influences both the gut–liver and the gut–brain axes at multiple levels [12].

This Special Issue explores the relationship between the gut microbiota and the host from multiple perspectives, and, despite the great number of studies which have been carried out, the network of interactions between GM signature and host functions still remains intricate and yet to be fully elucidated. Further studies are needed to resolve the current questions, since, in the era of precision medicine, GM could potentially revolutionize the development and prognosis of human diseases.
